# Parents’ Perspectives of Family Engagement with Early Childhood Education and Care During the COVID-19 Pandemic

**DOI:** 10.1007/s10643-022-01376-5

**Published:** 2022-08-05

**Authors:** Penny Levickis, Lisa Murray, Lynn Lee-Pang, Patricia Eadie, Jane Page, Wan Yi Lee, Georgie Hill

**Affiliations:** 1 Melbourne Graduate School of Education, The University of Melbourne, 3010 Ground Floor Kwong Lee Dow Building, 234 Queensberry St, Melbourne, VIC Australia; 2 Genetics, Murdoch Children’s Research Institute, Melbourne, Australia

**Keywords:** Early childhood education and care, COVID-19, Family engagement, Educator-parent partnerships

## Abstract

The COVID-19 pandemic has created significant challenges for Early Childhood Education and Care (ECEC) services and families, impacting family access to services and their communication and engagement with educators. This study aimed to examine parents’ perspectives of family engagement with ECEC services during the pandemic. Primary caregivers in Victoria at the time of recruitment (September–November 2020) were invited to participate. Of the 66 participants who completed an online survey, 25 also took part in semi-structured video call or phone interviews; qualitative findings from these interviews are reported in this paper. Four key themes were conceptualised using a reflexive thematic approach: (1) disruptions to ECEC access and attendance impacting on family routines and relationships, and child development; (2) barriers to family engagement; (3) ECEC educators’ support of families and children during the pandemic; and (4) increased parental appreciation of the ECEC profession. Findings revealed that disruptions to ECEC access and routines during the pandemic adversely impacted family engagement, and child learning and social-emotional wellbeing for some families. These were aggravated by other stressors, including increased parental responsibilities in the home, financial and health concerns, and changed work conditions. Findings also demonstrated successful methods used by educators to maintain communication and connections with families. Importantly, parents expressed increasing appreciation of the profession and an increased awareness of the value of family involvement in children’s learning. Learnings regarding strategies for effective and alternative ways of engaging families are discussed.

## Introduction

Although the health risk of exposure to the coronavirus disease is relatively low for young children (Bhopal et al., 2021; Sinha et al., 2020), responses to the pandemic may result in long term negative effects on children’s wellbeing and development. Mitigation measures of confinement and social distancing have been shown to reduce transmission of the disease, but these pandemic responses have often created stressful family environments, with potentially detrimental short- and long-term impacts on children and their families (Goldfeld et al., 2022; Schmeer et al., 2021). A critical component to promoting children’s resilience during adverse or traumatic events is for children to experience sensitive, responsive caregiving from the important adults in their lives (Bartlett & Vivrette, 2020). Quality early childhood education and care (ECEC) can provide a buffer against stressful home environments, particularly for those children experiencing less sensitive and responsive adult interactions in the home (Berry et al., 2016; Davies et al., 2021). Thus, during a pandemic it is feasible that ECEC settings may further protect children against the potential negative impacts of home isolation and unstable home environments.

### The Importance of Parent Engagement with ECEC and Family-Educator Partnerships

Children develop and learn within the contexts of the home environment, ECEC services, and community. The home environment, which includes the nature of parent-child interactions in and around the home, is an important predictor of child learning and development (Lehrl et al., 2020). Positive parenting behaviours and quality parent-child relationships can strengthen family bonds and support child learning and wellbeing (Perrin et al., 2016). In addition, strong partnerships between early childhood educators and families can boost parents’ confidence and capability to support young children’s learning. A recent meta-analysis demonstrated that educators’ use of practices of engagement (e.g., sending home information about the child) and parent engagement in the ECEC centre (such as volunteering) were indirectly associated with kindergarten academic readiness through increases in the quality of parent engagement in home learning activities (Barnett et al., 2020). Strong parent-educator relationships can be particularly important for disadvantaged families; for example, a study involving low-income parents and their preschool children demonstrated a positive association between parents’ perception of parent-educator communication, and the home learning environment (Lin et al., 2019). In Australia, the importance of strong family-educator partnerships is also reflected in policy frameworks such as the Australian Government’s Early Years Learning Framework (EYLF). The Framework describes the principles, practices and outcomes that support and enhance young children’s learning and states that partnerships with families “*are based on the foundations of understanding each other’s expectations and attitudes and building on the strength of each other’s knowledge*” (EYLF, 2009, p.13).

Building educator-family partnerships is especially important during stressful global events when parent-child relationships may be compromised. However, risk mitigation measures during the COVID-19 pandemic meant that ECEC services faced numerous communication challenges, such as reduced opportunities for face-to-face contact with children and families. While the pandemic resulted in the increased use of digital forms of communication – which research suggests can be beneficial in terms of making the work of educators and children’s learning more visible to families (Oke et al., 2021) - there are limitations to the use of online learning for very young learners (Hu et al., 2021), as well as the issue of the digital divide, whereby certain groups are at risk of being excluded from this form of interaction (Barnett et al., 2021; Shaik, 2022). Further, in an Australian qualitative study with educators during the pandemic, educators reported that digital technology was important for keeping services and families connected during the pandemic, but this was contingent on families having access and capacity to be online (Murray et al., 2021).

While the importance of building strong family-educator partnerships is clearly articulated in research and policy documents, recent evidence suggests that Australian educators may require increased support and training to improve their skills and confidence in working with families (Murphy et al., 2021). Gaining a better understanding of parents’ perspectives of engagement with ECEC and what they view as facilitating factors and barriers to engagement, may inform improvements to family engagement practices, thereby enhancing family–ECEC relationships, and supporting children’s learning and development across home and ECEC settings (Vuorinen, 2021).

### Theoretical Frameworks and Defining Family Engagement with ECEC

The current study is informed by Bronfenbrenner’s ecological model, whereby child development is viewed as a complex system of relationships affected by multiple levels of the child’s surrounding environment, from the immediate context of family and ECEC/school to broad cultural values, laws, and customs (Bronfenbrenner, 1977); Guy-Evans, 2020). Within this system, it is the microsystem, which includes the immediate environmental surrounds of the child such as family and early childhood education, that is thought to be the most influential (Bronfenbrenner & Ceci, 1994; Guy-Evans, 2020). In this way, children’s development is influenced by the interactions between the child and their primary caregivers (family and educators), but also by the connections and partnerships between these caregivers.

This study is also informed by Kim and Sheridan’s family engagement model (Kim & Sheridan, 2015). Two central dimensions of family engagement proposed by Kim & Sheridan (2015) include: (1) the structural dimension (ECEC and family efforts working side by side to support children’s learning and development); and (2) the relational dimension (a focus on continuing communication, connections, and interactions between families and educators) (Jeon et al., 2020). The structural dimension can include parent involvement in activities in ECEC settings, which may provide the opportunity to observe practices used by educators to promote children’s learning and development which parents can then implement in the home (Barnett et al., 2020). Communication is an important aspect of the relational dimension of family engagement and includes educators’ practices and strategies used to engage families (Eadie et al., 2017; Christenson, 2003).

### The Current Study

This qualitative study aimed to explore parents’ experiences of engagement with ECEC services during the COVID-19 pandemic in Australia. The State of Victoria went into Stage 3 lockdown on July 8, 2020, with Metropolitan Victoria moving into Stage 4 lockdown on August 2, 2020, which resulted in ECEC services being closed to all families except essential workers and vulnerable children for a period of two months (noting this lockdown lasted almost 16 weeks in total). This provided a unique opportunity to capture families’ experiences at a time when many families had limited access to ECEC and services were required to navigate alternative methods of engagement. The study aimed to examine parents’ varying experiences engaging with ECEC services, and to explore the challenges, successes, and learnings to carry forward.

The specific research questions were:

1. What was the impact of the pandemic on ways families engage with ECEC services?
2. What were parents’ perceived facilitating factors and barriers to engagement with ECEC services during the pandemic?
3. How did ECEC services support families and children with learning at home during the pandemic?

## Methods

### Participants

Participants who had taken part in an online survey examining family engagement with ECEC during the COVID-19 pandemic were invited to take part in this study. Survey participants were recruited primarily via Facebook and the research team’s ECEC networks (September-November 2020). Primary caregivers of young children (aged 1–6 years) living in Victoria, Australia who were enrolled in ECEC services at the time of recruitment were invited to take part. Upon completion of the online survey, participants were asked if they would be willing to take part in a one-to-one interview to further discuss their experiences of engagement with ECEC during the pandemic. Of the 66 participants who completed the survey, 37 parents (56.1%) agreed to be contacted for an interview. For the purpose of this paper, we are reporting on the qualitative data captured through the interviews. All participants were provided with a participant information statement and provided informed consent. Ethical approval for the study was obtained from the University of Melbourne Human Research Ethics Committee (#2057564). 

### Data Collection

Parents who agreed to take part in an interview were contacted via email and given the option of doing the interview by phone or video call (Zoom). Face-to-face was not an option due to being in Stage 4 lockdown. A topic guide of interview questions was used by the interviewer, which included prompts to encourage more detailed responses from parents. Table 1 highlights the key areas and example questions covered by the topic guide. All interviews were audio recorded with participant consent.

**Table 1:** Interview Topic Guide

Topic guide key area	Example interview questions
ECEC attendance and involvement during the pandemic	• How frequently was your child attending ECEC before the pandemic and how has this changed since the first lockdown in March? • What involvement have you had with your ECEC service and educators during the pandemic? How have you felt about this?
COVID-19 impact on ECEC-family communication	• What types of communication methods, for example telephone or email, has your ECEC service used with you and your family during the pandemic? - In what way, if any are these communication methods different from before the pandemic? - Which communication methods do you find most useful? - What type of information has ECEC been communicating with you during the pandemic, e.g., about fees, health and safety, individual information about your child, story time?
ECEC support for children’s learning and development	• Has your ECEC service provided any resources or assistance to help you support your child’s learning at home during the pandemic, for example ideas on activities to do at home? - If **yes**, what resources have they provided and have you used them? How have you used the resources? How useful have you found these resources? - If **no**, what resources or communication might have been helpful to support your child’s learning during lockdown?
COVID-19 impact on ECEC expectations and connectedness	• What, if any, expectations have your ECEC service or educators placed on you in relation to your child’s learning at home? • Have you had any expectations of your ECEC service in terms of supporting your child’s learning and development during the pandemic, and if so what are they? • Do you think your perception of and connectedness to ECEC is different during the pandemic compared to pre-pandemic?
COVID-19 impact on family relationships	• Has the pandemic impacted on your child’s interactions or connections with other family members, for example not seeing grandparents or other family members who had spent regular time with your child prior to the pandemic? • Has the pandemic impacted on your relationship or interactions with your child, and if so, in what way?

### Analyses

A reflexive thematic approach was used to analyse interview transcripts (Braun & Clarke, 2019). The approach was inductive (not hypothesis driven), semantic (focused on what parents are saying rather than trying to determine the assumptions underpinning what they are saying) and critical realist (focused on reporting an assumed reality evident in the data, i.e., reporting experiences, meanings and the reality of participants).

The first author began with data familiarization, reading and re-reading each transcript and noting initial impressions. Next, initial codes generated by the first author were refined by the second and third authors before identifying initial themes. Themes were conceptualised from clustering similar codes together and organised around a central concept or idea to capture shared meaning. To ensure analytic rigour, the thematic analysis process encompassed multiple interpretations of the data from people with diverse expertise with themes progressively refined via discussion and consensus amongst the lead researchers.

## Results

Of the 37 survey participants who consented to an interview, 25 completed a one-to-one interview with the first author. All participating parents were mothers. On average, parents who took part were from less disadvantaged socioeconomic areas and most parents had completed an undergraduate or postgraduate degree. Children of participants ranged in age from 13 months to five years and attended a mix of long day care services and stand-alone kindergartens, with one child attending a family day care service.

Four key themes were conceptualised from analyses of the interview data: (1) impact of disruptions to ECEC access for children and families; (2) barriers to family engagement during the pandemic; (3) ECEC supports for children and families during the pandemic; and (4) increased parental appreciation of the ECEC profession.

### Theme 1: Impact of Disruptions to ECEC Access for Children and Families

#### Disruptions to ECEC Access and Differing Parental Responses

The COVID-19 pandemic and associated mitigation measures across Victoria led to multiple changes to families’ ECEC access and attendance. While parents reported a range of experiences, a key issue identified was the disruption to family routines caused by changes in families’ access to ECEC services and personal factors brought on by the pandemic (e.g., changes to employment arrangements, such as working from home).

The multiplicity of contextual factors affecting each family had implications for their capacity to engage with ECEC. Parents who experienced multiple disruptions to family routines, which were compounded by a lack of other external supports for childcare (such as family members), responded with an increased awareness of their need for ECEC. These parents were often juggling multiple responsibilities (such as fulltime care, remote learning for children, and working from home) and reported increased stressors brought about by mitigation measures: “*we both were working full time the whole time, and you’re trying to fit eight hours of working a day when your child’s awake from 7:00 till 8:00 which means you’re working overnight*” (P4). For another parent, prolonged lack of ECEC access had a negative impact on their attitude towards engaging in activities with their child: “*But after the eight-week lockdown, I just do not want to do much more role playing. I’m just so over it. I think she sees the strain there, where at the beginning it was really fun and cute and now it’s just really annoying*” (P17).

Conversely, some families reported being much less impacted by mitigation measures and restricted access to ECEC. For these parents, factors such as living in a rural area which was not as greatly impacted by ECEC restrictions, as well as having childcare support from external family members contributed to less disruptions to routines and families’ experiences: “*Because we couldn’t access childcare, he was spending more time with my mum… he’s gotten to spend more time with her when he normally wouldn’t*” (P13). Despite differences in parental experiences and responses to the impact of disruptions during the pandemic, an overall effect observed similarly across all parent participants was increased parental stress.

#### Parent Concern for Children’s Wellbeing and Development

Another impact of changes to ECEC access and attendance during the pandemic was concern amongst parents about children’s social-emotional wellbeing, learning, and development. While some parents voiced concern more generally about children missing out on learning and preparation for school transition, the most common area of concern was children’s social-emotional development. Parents relayed concerns about the impact of social isolation on their child and the lack of opportunities for interactions with peers, both at home and in ECEC services (with reduced numbers of children in attendance). One parent described how their child “*cries because none of his friends are there*” (P10).

Some parents also reported concerns about the impact of reduced contact with wider family members for children during the pandemic. As described by one parent, the child *“just didn’t understand where people had disappeared to”* and *“essentially, all her relationships with family members became screen-based and missing out on the physical contact that you would otherwise have with a little kid”* (P23). Related to this concern were parents’ comments about how children were not engaging well with electronic means of communication with family members and friends. Children were described as *“want(ing) to run around, not participate in the conversation”* (P18), or being *“sick of it and just, ‘I want to see grandma in real life. I don’t want to talk on the iPad”* (P25).

Parents also expressed concerns about children’s emotional adjustment when they returned to ECEC services after a period of non-attendance due to the pandemic. One parent reported a regression in their child’s social skills:

*If we were at the playground and him and another kid were going to the swing at the same time, he’d get very aggressive, which is not like him at all. . the social anxiety of leaving has sort of started up again for both kids. . I don’t think it’s very great to just be relying on me, I like that they rely on other adults and stuff as well.* (P9)

Other parents reported their children’s disinclination and anxieties about returning to ECEC:

*At this age, it’s crucial to be socialising physically with kids…I do worry a little bit that he’s going to be super anxious about going back into large groups again because we’ve been at home for so long.* (P1)

#### Impact on Family Relationships

A positive effect that emerged from disruptions to ECEC attendance was strengthened sibling and parent-child relationships for some families. In some cases, forced home isolation led to parents appreciating the amount of quality time they had with their child; “*it’s probably strengthened our relationship and something I needed…as a mum to be like, okay, they’re only little once, stop going, stop trying to do everything*” (P11). Some parents also commented on the bonding observed between siblings from more time spent at home. One parent commented:

*I think it’s lovely the way my kids have bonded. They have just become little best friends just because of the amount of time they spent together* (P10).

While some parents felt the restrictions had a positive impact on their parent-child relationship, it is important to acknowledge that for other parents the stress of juggling working from home with caring for and home-schooling children was extremely challenging. This led to some parents feeling guilty for not investing in quality time with their children due to being occupied with other responsibilities: “*Instead of being able to enjoy our time together, I feel like we were always in this sort of half on, half off zone… I feel like the kids would have felt like we were always preoccupied, which we probably were* (P22).

Another parent observed that the additional stressors caused by the pandemic had led to increased parental stress, with negative impacts on the parent-child relationship: “*I actually find I’ve probably got a lot less patience with her now. I find myself getting more frustrated with her and a lot more short with her than I think I otherwise would have*” (P12).

### Theme 2: Barriers to Family Engagement During the Pandemic

#### Reduced or Removed Opportunities for Face-to-Face Contact Between Families and ECEC Services

A major barrier to families’ engagement with ECEC services during the pandemic was the elimination of opportunities for informal face-to-face conversations and parent observations of their child within their ECEC environment during pick-up and drop-off times. These opportunities were valued by parents and described as the “*main way we used to engage*” (P5). Face-to-face interactions were also key to providing parents with an increased understanding of their child’s experiences at ECEC services as well as their sense of community. However, as lamented by one parent:

*It went from in the yard pick up, they’re all running around and to have a bit of a chat. . there’s parents hanging around. . to suddenly having zero information... because you can’t see into the room, you can’t see anything so just have no sense of, it’s like a black hole that you’re sending child in.* (P5)

While some parents recognised that measures to limit face-to-face interactions were a constraint of the pandemic for ECEC services, other parents reported experiences of limited engagement with ECEC educators once attendance had resumed. One parent stated, “*it’s become a contactless, transactional kind of point*” (P14), while another pointed out:

*There’s a lot less interaction now. Again, that’s contributing to not knowing the carers as well. You know the key ones, who are the room leaders or whatever, but the other staff that you used to chat with particularly at the end of the day, to get to know them, you’re not getting that chance because you can’t hang around.* (P24)

Reduced opportunities for information exchange and parental engagement during face-to-face conversations with educators highlighted the need for services to find alternative ways to maintain these conversations with families during the pandemic. However, there was variability in services’ capacity to pivot to online modes of communication, with some services more reliant on face-to-face communication. As articulated by one parent:

*It is a culture of in-person communication, which is great when it’s working but pretty much impossible now. I think one of the challenging things they do with our service, is that they tend to do a lot of face-to-face communication, which is really nice. . But they’re not very digital… we would quite like them to pivot to something like Storypark now because otherwise we have so little assessment of the day.* (P8)

Changes to drop-off and pick-up routines often meant parents were not able to enter their child’s ECEC room to assist in settling them during the pandemic. This too, had negative implications for family engagement. As one parent described:

*It was harder for me. . because I was used to going into the room with him and getting him a little bit settled, and then sort of waiting five, ten minutes and then leaving, but to sort of have to hand him over at the door and he’s obviously in distress.* (P18)

#### Loss of Connections with Familiar Educators

Changes to attendance at ECEC services created barriers for some children and families in accessing or maintaining connections with the educators with whom children were most familiar. This loss of familiar relationships had a negative impact on family engagement and child wellbeing:

*I think it would’ve just been nice if he could’ve spoken to the two educators that he’s most connected with. . having some kind of connection with them would have been nice, even like a recorded video that they could just send home of them doing something in the room.* (P3)

The negative impact of lack of contact with services was felt most keenly by vulnerable families. In the case of one family of a child with a disability who experienced decreased ECEC attendance, feelings of disconnection and exclusion were reported:

*It was hurtful to be forgotten about, or to have had no contact from them, and to not receive any resources or help or contact for such a long period of time that we no longer felt part of the community and it’s hard not to see, that it’s just yet, another example of subtle discrimination and exclusion that people with disability experience…. .* (P23)

Changes to room groupings, staff rotations, and turnover during the pandemic also disrupted children’s connections with familiar educators, negatively impacting on their wellbeing. One parent commented on the child’s response to changes in room groupings and educators: *“It’s been harder. There’s days where he says, ‘Oh, I don’t want to go to childcare. I just want to stay home with you’”.* (P25)

#### Loss of Opportunities for Interactions with Other Families

Families reported loss of community and opportunities to interact with other families at their service, creating another barrier to ECEC engagement. As one parent commented: *“that’s kind of lost now. You don’t really get to speak to many parents anymore”.* (P11) Another parent described *“struggling”* in the *“new normal. . where you don’t get to be a part of a community as much”* (P8), while another reported the need to establish alternative modes of communication to maintain a sense of community: *“we are going to have to think about ways to change the methods of communication. I guess, to adapt to the fact that we don’t really get to talk to the parents very much anymore”.* (P8)

A phenomenon that emerged from the loss of opportunities for face-to-face interactions was the increase in families’ use of digital modes of communication, with several being organised by families. One parent described connecting with other families at their ECEC service through parent-initiated online modes:

*We’ve got a WhatsApp parent group too so there’s a lot being exchanged, ideas. . and we set-up a lot of joint phone calls. . they had some Zoom parties, everyone getting on and listening to a story and then that sparked its own set of connections”.* (P5)

### Theme 3: ECEC Supports for Children and Families During the Pandemic

#### Differing Levels and Varying Types of Support

The levels of support for home learning and approaches to communication with families varied widely across ECEC services, creating diverse experiences for families. While some parents reported receiving regular communication and “check-ins” from their ECEC service (e.g., emails, newsletters, and phone calls), online learning sessions (e.g., digital reading sessions), and resources for home learning (e.g., craft and learning activity packs for children to complete at home), other parents reported receiving little or no contact or resources from their service. The diversity in the levels and types of support parents received during the pandemic may reflect the differing ways ECEC services coped with the challenges of the pandemic, their digital fluency, and the differing infrastructure, funding and supports available to services. While some services appeared to be able to pivot relatively quickly to using remote modes of engagement, for other services, it was evident that the transition to digital modes of communication and learning was a major challenge. For example, one parent commented that staff at her ECEC service lacked confidence in *“writing and using computers and everything”.* (P23)

The varying levels and types of support provided by ECEC services for home learning also had mixed degrees of participation and usefulness for families. While some families welcomed and engaged with the home learning resources provided by their service, other families were less engaged due to reasons such as inconvenient timing (P13), a general lack of parent time (P15), and an inability to access resources required to do the activities provided by their ECEC service. However, there were other parents who reported benefiting from ideas and strategies provided by ECEC services via digital platforms:

*The watercolors, that was amazing, because I haven’t actually tried that. She absolutely loved it. So that was actually life changing. She does watercolors a lot now because of that.* (P17)

### Personalised, Two-Way Communication to Support Home Learning

Parents also reflected on the effectiveness of various strategies for remote communication and home learning. Successful ways of maintaining communication with families to promote family engagement and support home learning included: sending regular emails; making phone calls or video calls to see how families and children were doing; posting photos, videos and stories of what was happening at ECEC services via digital platforms such as “Storypark” or “Facebook”; posting videos of educators reading to children or online ‘live’ Zoom reading sessions; organising communal online events (e.g., virtual sessions for celebrating Father’s day); using apps for notifying parents about home learning activities planned by educators (e.g., “EDUCA”) and posting activities for home learning on online platforms (e.g. “Explore”); sharing learning and development plans with parents; and sending home resources and activity packs for children.

One parent’s description of positive virtual family engagement highlights the importance of regular contact and personal interactions, as well as establishing clear expectations:

*They conducted weekly Zoom sessions in small groups. They provided videos and tips and suggestions of activities every day. . they had a phone call with the parents at the start of each lockdown period to explain to us what was going to happen, to listen to any concerns…just to set the tone and make sure we knew what the expectations were at each side… We had an app that we were communicating through—ClassDojo—so they would post tasks or videos or suggestions, or just have regular contact daily. And then they would organize small group sessions of around three kids plus the two educators once a week for about 25 min. . I felt there was a really strong connection there and we felt very informed, and it was just like virtual kinder basically.* (P25)

Overall, strategies that were successful in engaging families remotely and supporting children’s learning from home were those that that focused on fostering genuine connections with families and children, rather than relaying information. Communication with families that was regular and timely was well-received by parents, as well as open communication channels, whereby educators remained accessible by phone and digital modes to families, adopting two-way modes of communication. One parent (P24) described how the ECEC service offered parents the ability to book Zoom meetings with educators, while another parent commented on the benefits of the *Dojo* app, which appeared to enhance communication and engagement, both during and post lockdowns:

*I think in a way the kinder got dragged into the virtual environment out of necessity, but it’s been fantastic now that they’re back to learning face-to-face, they’re still using it to send us pictures of what they’re doing, which is lovely and to communicate… I find it really simple and direct and much more easy for me as well as a parent who was often not around to pick up and drop off…now I feel like I know what’s going on a bit more at kinder.* (P25)

On the other hand, less effective strategies for remote communication and home learning included: generalised emails, newsletters, and phone calls from centres about policy/health and safety updates which lacked a personal touch; adult-focused phone calls which were either not purposeful or at a busy time for parents; home resource packs that were not engaging, personalised or authentic for children (P1); and videos of educators reading to children in the centre for families to watch at home. For instance, one parent (P20) described how it was difficult to hear or see the teacher as she read to the children who were onsite, as the video recording would be disrupted by the sounds of other children in the room. Videos of educators demonstrating activities that parents could replicate at home with children were also met with limited success. One parent stated:

*We did do activities with our kids but I didn’t have a lot of patience, unless they seemed like they would be really easy and absorbing. . it’s not the same environment as the daycare centre. . they just behave differently at daycare, especially with the activities.* (P8)

#### Challenges of Engaging Young Children Through Digital Modes of Learning

One of the key challenges to ECEC engagement during the pandemic identified by parents was the difficulty in engaging young children through digital modes of learning: “*It’s that digital thing, like it’s hard to engage with a screen. . she didn’t really participate as much as I think she does when she’s in person”* (P12); *and “she doesn’t enjoy the remote, the skyping. . She’ll get into it for ‘Hi. Yay, I get to see your face’ kind of thing, and then they try and read her a story, but she’s just not into it. . She wants the physical touch of them”.* (P11) Parents’ comments in relation to children’s lack of interest in remote learning and preference for physical, face-to-face activities reflected the importance of the close physical proximity of educators for young learners.

### Theme 4: Parental Appreciation for the ECEC Profession

Some parents reflected that the pandemic had highlighted the work of educators in not only providing care for children but also playing a key role in supporting children’s learning and development, acknowledging “*a deep appreciation for the fact that they’re educators, not babysitters*”. (P4)

#### Appreciation of Educators’ Efforts to Provide Ongoing Support to Families and Children

Parents reflected on their appreciation for the lengths that educators had gone to in supporting families and children during the pandemic, particularly given the pressures on the sector. Parents also expressed gratitude in relation to educators’ commitment to ensuring the health and wellbeing of children, as well as their families: “*Throughout the whole situation, they’ve always, always come back to saying that their primary concern is about the welfare of not just the children in their care at the present time, but the welfare of families who have been really seriously impacted by the pandemic.”* (P6)

#### Benefits of ECEC for Families and Children

In some cases, additional efforts by educators to connect with families during lockdowns increased the visibility of children’s learning and parents’ engagement in their child’s learning at home. One parent described the value of observing the educator’s interactions with her child during a Zoom call:

“*I was sitting in on his Zoom calls and I knew they were learning about opposites. When we were reading a book later, I could say, “Oh, there’s the ‘up’ shape. What is the opposite of the ‘up’ shape? Oh, it’s the down shape.“ You could filter that learning into your playtime. I wouldn’t have known that he’d just learnt that concept if I hadn’t been around and had that communication.* (P25)

Acknowledging the important contribution educators make to supporting children’s learning and development, another parent noted, *“We didn’t think that we could match what the educators were offering in terms of, I guess, really considered learning experiences”.* Some parents reflected not only on the importance of ECEC for their children’s “*learning and discovering”* (P1) but also spoke about the benefits of ECEC for their own wellbeing: *“they went back and it was good for me. It was good for them… my mood and my productivity was so much better.”* (P11)

#### Parental Acknowledgement of Challenges Faced by the Early Childhood Profession

Parents’ appreciation of the ECEC profession was reflected in their comments acknowledging the value of ECEC in society, and the challenges faced by the sector during the pandemic: “*I think it’s been incredibly tough, and I really hope that at a policy and a government level, there could be some recognition of the fact that this service, the sector is the absolute lifeblood of our society.*” (P4) Another parent acknowledged that it is *“one of the most difficult professions to be in right now.”* (P1) Parents also noted that despite the immense challenges and stress faced by educators, educators demonstrated dedication to supporting the children and families in their care.

## Discussion

The current study used a qualitative approach to explore family engagement with ECEC during the COVID-19 pandemic from the perspectives of parents living in Victoria during one of the state’s strictest lockdown periods. In addressing the first research question relating to the impact of the pandemic on the ways in which families engaged with ECEC services, findings revealed that in many cases, disruptions to ECEC access and routines adversely impacted family engagement and parents’ views of their child’s learning and development, in particular, children’s social-emotional wellbeing. These were aggravated by other stressors, including increased parental responsibilities in the home and changed work conditions. The adverse impacts observed by parents on children’s social-emotional wellbeing were thought to be due to a lack of peer interactions and isolation from ECEC daily interactions, resulting in a dependence on immediate family members. Recent studies have reported the impact of the pandemic on children’s social-emotional wellbeing due to a lack of opportunity for social interactions (Stites et al., 2021; Egan et al., 2021). Evidence suggests that ECEC settings can provide children with increased opportunities for social-emotional learning and strengthen kindergarten/school readiness via responsive, sensitive daily educator-child interactions (Blewitt et al., 2018). Interactions with peers from an early age have also been identified as important for social-emotional development, with research demonstrating that social competence and positive relations with peers from an early age may be protective against later psychosocial problems (Egan et al., 2021; Hay, 2005).

Despite the negative impacts of the pandemic mitigation measures on social interactions, the increase in quality time spent at home allowed for some families to strengthen their family bonds, in terms of both sibling and parent-child relationships. It may be that protective factors, such as employment and financial stability, and good health, contributed to some families having positive experiences during the stressful periods of lockdowns. However, this also highlights that for those who were already experiencing socioeconomic hardship and vulnerability, there was an increased risk of mental health problems and strained family relationships (Evans et al., 2020), underscoring the importance of access to ECEC and family support services during the pandemic.

A major impact of the pandemic on how ECEC services engaged with families was the need for services to find alternative ways of communicating and connecting with families, outside of traditional face-to-face methods of engagement. Indeed, a major barrier to engagement with ECEC (research question 2) for many families was the loss of opportunities for face-to-face conversations with educators, especially when digital forms of communication were a challenge to implement or had not been widely used prior to the pandemic. This proved particularly challenging when a major strength of an ECEC service was delivering face-to-face communication, but this form of engagement was suddenly no longer feasible. Another barrier to engagement was the use of remote, online activities, which proved challenging with very young children. Face-to-face contact and interaction with educators and peers for very young children has been identified as critical to their learning and development (Barnett et al., 2021). Previous research exploring parental beliefs about online learning for young children reported parents believed that online learning is less effective than traditional early childhood education environments and lacks the social interactions required to engage young children (Dong et al., 2020). Educators have also reported that online learning lacks the interaction and play-based learning opportunities for young children, but creating online learning communities could enhance parent engagement with ECEC, enabling educators to support children’s learning and development in the home (Hu et al., 2021).

Importantly, findings also demonstrated successful methods used by educators to maintain communication and engagement with families, and support children’s learning and development at home (research question 3). Findings from this study showed that communication that is strongly focused on making genuine connections with families and children (e.g., maintaining established relationships with familiar educators) can promote family engagement. Adopting methods for two-way conversations with families, rather than one-way delivery of more general information was also more effective. This is in line with Kim and Sheridan’s (2015) family engagement model, where it is evident that for families and educators to work together to promote children’s learning and development, the relational dimension of engagement needs to be established, that is, continued communication, connections and interactions between families and educators. By establishing trusting relationships between parents and educators, continuity across the home and ECEC regarding children’s learning and development is more likely to occur. Findings also align with Bronfenbrenner’s ecological model (1977), whereby the connections and partnerships that occur within the microsystem, between family and early childhood educators influence children’s learning and development.

It is evident that successful family engagement approaches are built on strong relationships and tailored to meet the individual needs of children and families. However, it should also be acknowledged that this approach encompasses a number of challenges, not least the increased burden on educators, who are already experiencing high levels of burnout and poor wellbeing (Berger et al., 2022; Eadie et al., 2021). For educators to provide quality and consistent support for children and families, their own wellbeing needs to be supported. Therefore, effective family engagement approaches must account for educators’ wellbeing and needs as well as the needs of the children and families in their care.

### Limitations and Future Directions

This study has a number of limitations. First, participants were recruited via online advertising, so only those who have access to the internet could take part and participants were self-selecting. In addition, parents were all mothers, from English speaking backgrounds, from less disadvantaged socioeconomic areas and had completed high levels of education. Experiences of ECEC engagement during the pandemic may have been quite different for families experiencing socioeconomic disadvantage or from different language or cultural backgrounds. While technology use was highlighted as an effective strategy for communicating with families and supporting the home learning environment, this approach may create further barriers for families without digital access or capacity (Barnett et al., 2021; Murray et al., 2021). Further research exploring effective approaches for educators to build relationships with families who lack either the access or capacity to engage in digital forms of engagement is much needed, given the rise in the use of digital platforms in ECEC settings. While educators may feel knowledgeable regarding how to work with families, a major challenge lies in converting their knowledge into practice to improve partnerships with families (Murphy et al., 2021). Professional learning for educators that focuses on building effective, collaborative family partnerships and supporting the home learning environment should also be a priority.

The pandemic has shone a spotlight on the critical role ECEC plays, not only in providing care for children, but importantly, the vital role educators play in supporting children’s learning and development and parental workforce participation. Findings from this study show it does appear parents’ perspectives of the role of ECEC is shifting, with a greater understanding not only of the ways in which educators promote children’s learning and development but also the contribution that parents can make to children’s learning. This has implications for greater family involvement in children’s learning and experiences within ECEC programs. Government investment and public messaging is needed to further highlight the visibility of the vital work of educators and to enhance the value of the sector in society (Eadie et al., 2021).

## Conclusions

This study provides important insights into barriers and facilitators to family engagement with ECEC in the context of COVID-19, and learnings for strengthening family-ECEC partnerships beyond the pandemic. The pandemic has highlighted the importance of ECEC for families, as well as the need for ECEC services to work with families to find personalised methods of engagement that meet their varying contexts and needs.. However, to do this successfully, the sector needs to be supported and resourced to confidently use digital methods modes of communication, and to engage with families collaboratively to establish communication practices that foster personalized, ongoing, and authentic engagement.
